# Pre-COVID era pediatric disease incidence in Kazakhstan: regional panel data analysis of multiple disease groups (2010–2019)

**DOI:** 10.3389/fpubh.2025.1615521

**Published:** 2025-06-09

**Authors:** Nurlan Smagulov, Olzhas Zhamantayev, Aidar Aitkulov, Nurbek Yerdessov, Karina Nukeshtayeva, Zhanerke Bolatova, Zhyldyz Kurzhunbaeva

**Affiliations:** ^1^Research Park of Biotechnology and Eco-Monitoring, Karaganda Buketov University, Karaganda, Kazakhstan; ^2^School of Public Health, Karaganda Medical University, Karaganda, Kazakhstan; ^3^Faculty of Biology and Geography, Karaganda Buketov University, Karaganda, Kazakhstan; ^4^Department of Science and High Technology, University of Insubria, Varese, Italy

**Keywords:** pediatric morbidity, Kazakhstan, respiratory diseases, asthma, nervous system disorders, socioeconomic determinants, healthcare access, disparity

## Abstract

**Background:**

While child morbidity in Kazakhstan is studied, existing research often prioritizes mortality or infectious diseases over non-communicable conditions. This study fills this gap by examining socioeconomic, demographic, and healthcare factors linked to respiratory diseases, asthma, and nervous system disorders among children aged 0–14 years across Kazakhstan from 2010 to 2019 highlighting regional context.

**Methods:**

Panel data from 14 regions were analyzed using linear mixed models with autoregressive covariance to address regional and temporal heterogeneity. Log-transformed incidence rates of respiratory diseases (J00-J99), asthma (J45), and nervous system diseases (G00-G99) were modeled against predictors including GRP per capita, unemployment, population density, Gini coefficient, pediatrician density, and hospital resources. Other variables with variance inflation factors ≥5 were excluded to mitigate multicollinearity.

**Results:**

Respiratory diseases showed the highest mean incidence (57,329.86 per 100,000), with significant regional variation. Aqtöbe, Atyrau, and South Kazakhstan had 12–25% lower incidence compared to Zhambyl (reference), while Pavlodar and North Kazakhstan had 35–61% higher rates. A 1% increase in population density correlated with a 1.05% decrease in respiratory disease incidence (*p* = 0.008), whereas unemployment was linked to a 0.41% rise (*p* = 0.029). Asthma incidence increased by 140% over the decade, with higher rates in regions with greater income inequality (0.26% increase per 1% rise in low-income households, *p* = 0.032). Nervous system disorders showed limited associations, with unemployment as the sole predictor (0.69% increase per 1% rise, *p* = 0.040). Temporal trends revealed declines in most diseases, but neoplasms, diabetes, and asthma increased significantly.

**Conclusion:**

The study addresses the lack of localized socioeconomic and healthcare analyses for respiratory diseases, asthma, and nervous system disorders among children, providing evidence for region-specific policy interventions. Respiratory diseases and asthma among Kazakhstani children 0–14 years had associations with the regional economic conditions, healthcare utilization, and inequality. Population density and income inequality were consistent predictors, while nervous system disorders showed fewer clear associations. Our findings show distinct regional patterns in pediatric morbidity, linking health outcomes to localized socioeconomic and healthcare conditions.

## 1 Introduction

The well-being of a nation’s children serves as an important sign of its socioeconomic progress and a window into its future path. As earlier studies have noted, child health reveals not only the present state of public welfare but also hints at the adult population’s eventual condition, since early years form habits and risks that persist through life (1). Understanding the forces behind child health thus becomes necessary for designing policies to raise societal welfare. These forces span a wide range, combining biological traits with broader social and economic elements, such as family income, parental schooling, housing quality, and access to medical care, that together leave a deep mark (2, 3).

The age range of 0 to 14 years stands out for several reasons. This period covers key phases of physical, mental, and emotional growth, where early experiences, whether poor nutrition, unsafe surroundings, or emotional strain, can cast lasting effects on health. Children in this group also tend to be more affected by outside conditions than adults, making them a clear measure of society’s health gaps (4). Protecting their well-being calls for efforts that reach beyond medical care alone. It requires action from political leaders and cooperation across fields to not only address illness but also build settings that support healthy development (5).

Research on pediatric disease incidence rates is scarce in literature, with not many studies providing detailed breakdowns, particularly for non-communicable diseases (NCDs). Organizations such as the WHO and UNICEF often prioritize mortality data or focus on leading causes like infectious diseases and malnutrition, with less emphasis on NCDs or system-specific morbidity in children aged 0–14.

Globally, respiratory conditions, mainly asthma and pneumonia, are major contributors to morbidity. According to the study on asthma incidence among children 0–14 in Asia, it varies widely, with the highest in the Philippines (1,686.9 per 100,000) and the lowest in Bhutan (237.9 per 100,000) (6). Immune disorders, such as primary immunodeficiencies, are less quantified, but a U. S. study estimates a prevalence of 1 in 1,200 individuals (7). The incidence of type 1 diabetes among children under the age of 15 is increasing, with recent European data indicating an annual rate of 29.1 new cases per 100,000 population (8). Anemia affects approximately 40% of children under 5 (293 million in 2016), with urban prevalence often lower due to better nutrition but still significant in poor urban slums (9). A study from Kazakhstan found that real prevalence of asthma in Kazakhstan is estimated to be 5.33 times higher than official records, with many patients wrongfully diagnosed with COPD or obstructive bronchitis (10). Another study reporting the asthma incidence rate ranged from 67.5 to 185.9 among boys and 38.2 to 115.7 among girls per 100,000 population, with the highest rates observed in the 5–11 age group (308–351 cases) (11). Regarding nervous system disease trends in Kazakhstan, mild variability in national cerebral palsy incidence was observed in 2010–2019, ranging from 68.7 to 83.3 per 100,000 total population (12). Additionally, a significant increase in the incidence of epilepsy was documented, rising from 26.15 in 2014 to 88.80 in 2020 per 100,000 total population (13).

Child health measures, such as rates of illness, tie closely to differences in regional economic growth, environmental quality, and the strength of schools and healthcare systems in managing disease risks (14). Everyday factors, including food availability, home stability, caregivers’ working conditions, access to play areas, and the nature of schooling, can shape children’s health in distinct ways across the globe (15–17). Research by Cohen and others demonstrates that a child’s economic background can be associated with heart disease deaths and other specific causes later in life (18, 19). In certain areas, especially lower-income regions like Kazakhstan, unmet goals for improving child health have led to rising illness and death rates, underscoring the necessity for targeted initiatives (20, 21). Apart from socioeconomic factors, a Norwegian study showed that having more doctors in a local area is associated with better newborn outcomes, including fewer fetal deaths and higher birth weights, with this being independent of socioeconomic factors (22). In contrast, the shortage of doctors creates a significant barrier to delivering quality healthcare, intensifying disparities in health (23).

Central Asia, with Kazakhstan as a key example, offers a unique view of these patterns. Since gaining independence from the Soviet Union, the region has experienced serious economic and social transformations, resulting in significant changes in living conditions and health outcomes (24). The healthcare system in Kazakhstan has undergone significant reforms, optimizing health professional density, average days of stay at hospital, launching national screening programs, and yet gaps persist (25). Public expenditures on health care account for only 1.8–3.2% of GRP, covering just 58% of total expenditures, leading to significant out-of-pocket costs for patients, which account for approximately 38% of total healthcare expenditures, exceeding the WHO norm of 20% (26).

Our research question was “What is the relationship between socioeconomic, demographic, and healthcare indicators and pediatric disease incidence among children aged 0–14 years in Kazakhstan’s regions from 2010 to 2019?

The aim of our study was to identify statistically significant predictors of pediatric disease incidence across Kazakhstani regions using panel data regression analysis, accounting for regional and temporal heterogeneity. We also hypothesized that higher income inequality (Gini coefficient) would correlate with increased pediatric disease incidence due to disparities in healthcare access.

## 2 Materials and methods

### 2.1 Data collection and variables

We conducted a panel data analysis using data from the Bureau of National Statistics of the Republic of Kazakhstan, covering the period from 2010 to 2019. National statistics data undergoes standard validation procedures to ensure accuracy and consistency. The dataset comprised panel data pooled across 14 regions of Kazakhstan from 2010 to 2019, yielding 140 region-year observations, except for South Kazakhstan, which contributed data until 2017 (138 observations total) (Supplementary Table 1). The selection criteria included high incidence rates, significant variability across regions and time, notable trends, and public health relevance. Quantitative data on socioeconomic, demographic, and healthcare indicators were extracted. All variables used in our analysis were continuous, except for regions which were treated as a categorical variables. Incidence rates for children aged 0–14 years were collected from national and regional statistical reference books.

The study included the following socioeconomic, demographic, and healthcare variables based on prior literature and data availability (27–30):

- GRP per capita (thousand tenge) represents the gross regional product per person, reflecting the economic output of each region adjusted for population size.
- Population density (persons per km^2^) captures relative differences in settlement patterns across Kazakhstan’s regions, shaped by the country’s vast territory (2.7 million km^2^), rather than serving as a direct proxy for urbanization.
- Living wage (thousand tenge) denotes the minimum income required to meet basic needs, including food, housing, and healthcare.
- Unemployed population (thousand people) refers to individuals actively seeking work but without employment, serving as an indicator of economic instability.
- Average monthly nominal salary (thousand tenge) captures the mean gross earnings before tax deductions, reflecting regional income levels.
- Gini coefficient quantifies income inequality on a scale from 0 (perfect equality) to 1 (maximum inequality).
- Population with income below the living wage (%) estimates the proportion of residents earning less than the subsistence minimum.
- Housing provision (sq. m. per resident) indicates the average living space per person, a proxy for living standards.
- Marriage rate (per 1,000 population) records the annual number of marriages per 1,000 residents, reflecting social stability.
- Divorce rate (per 1,000 population) measures the annual number of divorces per 1,000 residents, indicating family structure dynamics.
- Average monthly nominal salary in healthcare (thousand tenge) assesses the earnings of healthcare workers, influencing workforce retention.
- Number of physicians (excluding dentists) per 10,000 population evaluates the density of practicing doctors, a key metric for healthcare access.
- Pediatricians per 10,000 population specifically measures the availability of child healthcare specialists.
- Obstetrician–gynecologists per 10,000 population tracks maternal healthcare capacity.
- Nursing staff per 10,000 population quantifies the nursing workforce, critical for service delivery.
- Nursing staff (midwifery) per 10,000 population focuses on midwives, essential for maternal and neonatal care.
- Number of hospital beds for children per 10,000 population assesses pediatric inpatient infrastructure.
- Average length of hospital stay (LOS) for children 0–14 years (days) indicates the mean duration of hospitalization for pediatric patients.

In our study we analyzed childhood disease incidence categorized by the International Classification of Diseases, 10th Revision (ICD–10). Below are detailed definitions of the disease groups, including specific conditions covered under each code (31):

- All diseases (A00–Z99) category represents the total incidence of all diagnosed conditions in children aged 0–14 years, covering infectious diseases, injuries, congenital anomalies, and chronic disorders. This category serves as a comprehensive measure of overall pediatric morbidity.
- Neoplasms (C00–D49) include both benign and malignant tumors, such as leukemia, lymphomas, central nervous system tumors, and benign neoplasms of the skin.
- Diseases of the blood and blood-forming organs (D50–D89) covers disorders such as iron-deficiency anemia, vitamin B12 deficiency anemia, hemolytic anemias, coagulation defects, and thrombocytopenia. Iron-deficiency anemia (D50) specifically refers to anemia caused by inadequate iron intake or absorption, including nutritional iron deficiency.
- Endocrine, nutritional, and metabolic diseases (E00–E90) include thyroid disorders, type 1 diabetes mellitus, type 2 diabetes mellitus, malnutrition, obesity, and metabolic syndromes. Diabetes mellitus (E10–E14) focuses on chronic hyperglycemia, subdivided into insulin-dependent diabetes, non-insulin-dependent diabetes, malnutrition-related diabetes, and other specified forms (E13–E14).
- Diseases of the nervous system (G00–G99) include conditions such as meningitis, epilepsy, cerebral palsy, migraines, and neurodegenerative disorders.
- Diseases of the circulatory system (I00–I99) include rheumatic heart disease, hypertensive diseases, ischemic heart disease, and cerebrovascular accidents.
- Diseases of the respiratory system (J00–J99) cover acute upper respiratory infections, pneumonia, asthma, chronic obstructive pulmonary disease, and bronchitis. Asthma (J45) specifically refers to chronic airway inflammation characterized by recurrent wheezing, breathlessness, and coughing.
- Diseases of the digestive system (K00–K95) include gastroesophageal reflux disease, peptic ulcers, gastritis, hepatitis, and inflammatory bowel disease.
- Diseases of the musculoskeletal system and connective tissue (M00–M99) cover juvenile idiopathic arthritis, osteomyelitis, osteoporosis, and systemic connective tissue disorders.
- Diseases of the genitourinary system (N00–N99) include acute glomerulonephritis, urinary tract infections, renal failure, and disorders of the male and female genital tracts.

### 2.2 Data analysis

Dependent variables (disease incidence rates) were classified using ICD-10 codes as reported by the Bureau of National Statistics, with incidence rates calculated per 100,000 children aged 0–14 years.

Log transformation was applied to both dependent and independent continuous variables to address right-skewness, stabilize variance, and interpret regression coefficients as elasticities, representing the percentage change in disease incidence associated with a 1 % change in each predictor. Since no zero values were present in the incidence data, no additive constant (e.g., +1) was required for transformation.

To identify disease groups warranting detailed analysis, we evaluated incidence rates for children aged 0–14 years across 14 regions in Kazakhstan from 2010 to 2019 (South Kazakhstan until 2017). The selection criteria included high incidence rates, significant variability across regions and time, notable trends, and public health relevance.

Descriptive statistics (mean, standard deviation, and coefficient of variation) were computed for all variables to assess both disease burden and predictor variability. Temporal trends were evaluated using line plots stratified by region. Spearman’s rank correlation (*ρ*) was employed to examine associations between log-transformed variables, as some predictors violated normality assumptions (Shapiro–Wilk *p* < 0.05). However, with panel data and sufficient observations (>100), normality is less critical due to the central limit theorem (32).

We applied linear regression analysis to examine temporal trends in childhood disease incidence across all regions of Kazakhstan from 2010 to 2019 and to quantify the average annual changes in the variables. Using regression coefficients, the analysis provided insights into both the magnitude and direction of changes in the disease burden (33).

A linear mixed model (LMM) with fixed effects for regions and years was employed to examine the proportional relationships between the log-transformed predictors and the incidence rates of respiratory diseases, nervous system diseases, and asthma. Prior to fitting the LMM, multicollinearity among the log-transformed predictors was assessed by calculating the Variance Inflation Factor (VIF) using a linear regression model in SPSS. Initial analysis revealed high VIF values for several predictors. We removed predictors that were theoretically redundant or less relevant to the research question, including ln (Avg monthly salary), ln (Living wage), ln (Marriage rate), ln (Divorce rate), ln (Salary healthcare), ln (Physician density), ln (Obstetrician-gynecologist density), ln (Nurse density), and ln (Midwifery density). This exclusion ensured model stability but may limit insights into healthcare workforce effects, potentially underestimating their role in disease incidence. A second linear regression with the remaining predictors [ln (GRP per capita), ln (Unemployed population), ln (Population density), ln (Gini), ln (Income below living wage), ln (Housing per resident), ln (Pediatrician density), ln (Hospital beds for children), ln (Length of stay)] yielded VIF values below 5.

After running the LMM, regression residuals were examined to verify model assumptions. Residual plots (residuals vs. predicted values) were generated to check for homoscedasticity and linearity, confirming that the residuals had no systematic patterns and were approximately homoscedastic, supporting the validity of the log-transformation and model specification.

All analyses were conducted in SPSS Version 26 (IBM Corp., Armonk, NY), with statistical significance set at *p* < 0.05.

## 3 Results

### 3.1 Descriptive statistics and trends

Summary statistics (Table 1) showed that respiratory diseases had the highest mean incidence (57,329.86 per 100,000, CV = 0.40), followed by digestive system diseases (5,898.30 per 100,000, CV = 0.41) and blood diseases (3,960.85 per 100,000, CV = 0.47).

**Table 1:** Summary statistics for diseases variables.

Disease group	Mean incidence (per 100,000)	SD	CV
All diseases (A00–Z99)	93,233.17	30,242.49	0.32
Neoplasms (C00–D49)	164.22	156.13	0.95
Diseases of the blood and blood-forming organs (D50–D89)	3,960.85	1,855.84	0.47
Iron-deficiency anemia (D50)	3586.24	1,794.04	0.50
Endocrine, nutritional, and metabolic diseases (E00–E90)	956.19	492.35	0.51
Diabetes mellitus (E10–E14)	9.97	5.09	0.51
Nervous system diseases (G00–G99)	2,834.57	1,188.05	0.42
Circulatory system diseases (I00–I99)	327.00	203.60	0.62
Respiratory system diseases (J00–J99)	57,329.86	23,112.98	0.40
Asthma (J45)	97.11	70.34	0.72
Digestive system diseases (K00–K95)	5,898.30	2,435.87	0.41
Musculoskeletal and connective tissue diseases (M00–M99)	944.56	637.53	0.67
Genitourinary system diseases (N00–N99)	1300.15	509.30	0.39

Table 2 shows a general decline in disease burden from 2010 to 2019 across most categories: Blood and blood-forming diseases; Iron-deficiency anemia; Endocrine, nutritional, and metabolic diseases; Circulatory system diseases; Respiratory system diseases; Genitourinary system diseases. Trend analysis showed that the largest annual average decrease was observed in iron-deficiency anemia at −213.68 (95% CI: −313.62; −113.74), followed by diseases of the blood and blood-forming organs at −234.58 (95% CI: −337.10; −132.06) and endocrine, nutritional, and metabolic diseases at −64.37 (95% CI: −91.43; −37.32), all statistically significant. Circulatory system diseases showed a decrease of −20.46 (95% CI: −32.02; −8.91), while genitourinary system diseases decreased by −51.40 (95% CI: −80.29; −22.51), both also statistically significant. In contrast, neoplasms increased annually on average by 10.99 (95% CI: 2.05; 19.93), diabetes mellitus by 0.59 (95% CI: 0.31; 0.88), and asthma by 10.03 (95% CI: 6.22; 13.82), all with statistically significant positive trends. Some categories, such as respiratory and digestive diseases, show wide confidence intervals, indicating variability in the annual average change estimates due to non-significant statistical differences.

**Table 2:** Trend analysis for disease incidence in Kazakhstan, 2010–2019.

Disease group	2010 Mean	2019 Mean	% Change (2010–2019)	Annual average change (95%CI)
All diseases (A00–Z99)	99,987.42	89,070.00	−10.92%	−910.9 (−2696.19; 874.44)
Neoplasms (C00–D49)	124.45	199.29	60.14%	10.994 (2.05; 19.93)*
Diseases of the blood and blood-forming organs (D50–D89)	5,023.14	2,969.10	−40.89%	−234.58 (−337.10; −132.06)**
Iron-deficiency anemia (D50)	4,515.99	2,649.35	−41.33%	−213.68 (−313.62; −113.74)**
Endocrine, nutritional, and metabolic diseases (E00–E90)	1,372.54	742.99	−45.87%	−64.37 (−91.43; −37.32)**
Diabetes mellitus (E10–E14)	6.79	13.51	99.03%	0.59 (0.31; 0.88)**
Nervous system diseases (G00–G99)	2,984.52	2,742.87	−8.09%	−8.29 (−78.67; 62.09)
Circulatory system diseases (I00–I99)	455.27	245.49	−46.07%	−20.46 (−32.02; −8.91)**
Respiratory system diseases (J00–J99)	57,986.56	56,602.65	−2.39%	−42.49 (−1412.01; 1327.02)
Asthma (J45)	59.15	142.08	140.20%	10.03 (6.22; 13.82)**
Digestive system diseases (K00–K95)	6,468.34	5,887.87	−8.98%	8.53 (−135.79; 152.86)
Musculoskeletal and connective tissue diseases (M00–M99)	1,104.11	1,010.88	−8.44%	−7.57 (−45.33; 30.18)
Genitou*rinary system d*iseases (N00–N99)	1,616.85	1,101.39	−31.88%	−51.40 (−80.29: −22.51)**

*p-value < 0.05, **p-value < 0.001.

Based on these findings, we selected respiratory diseases for further analysis due to their high mean incidence (57,329.86 per 100,000) and moderate variability (CV = 0.40), making them a serious public health concern with potential socioeconomic and demographic drivers. Nervous system diseases were chosen despite a slight decline in incidence, given their relatively high incidence (2,834.57 per 100,000), variability (CV = 0.42), and relevance to child neurodevelopment, which may be influenced by socioeconomic factors.

**Figure 1:**
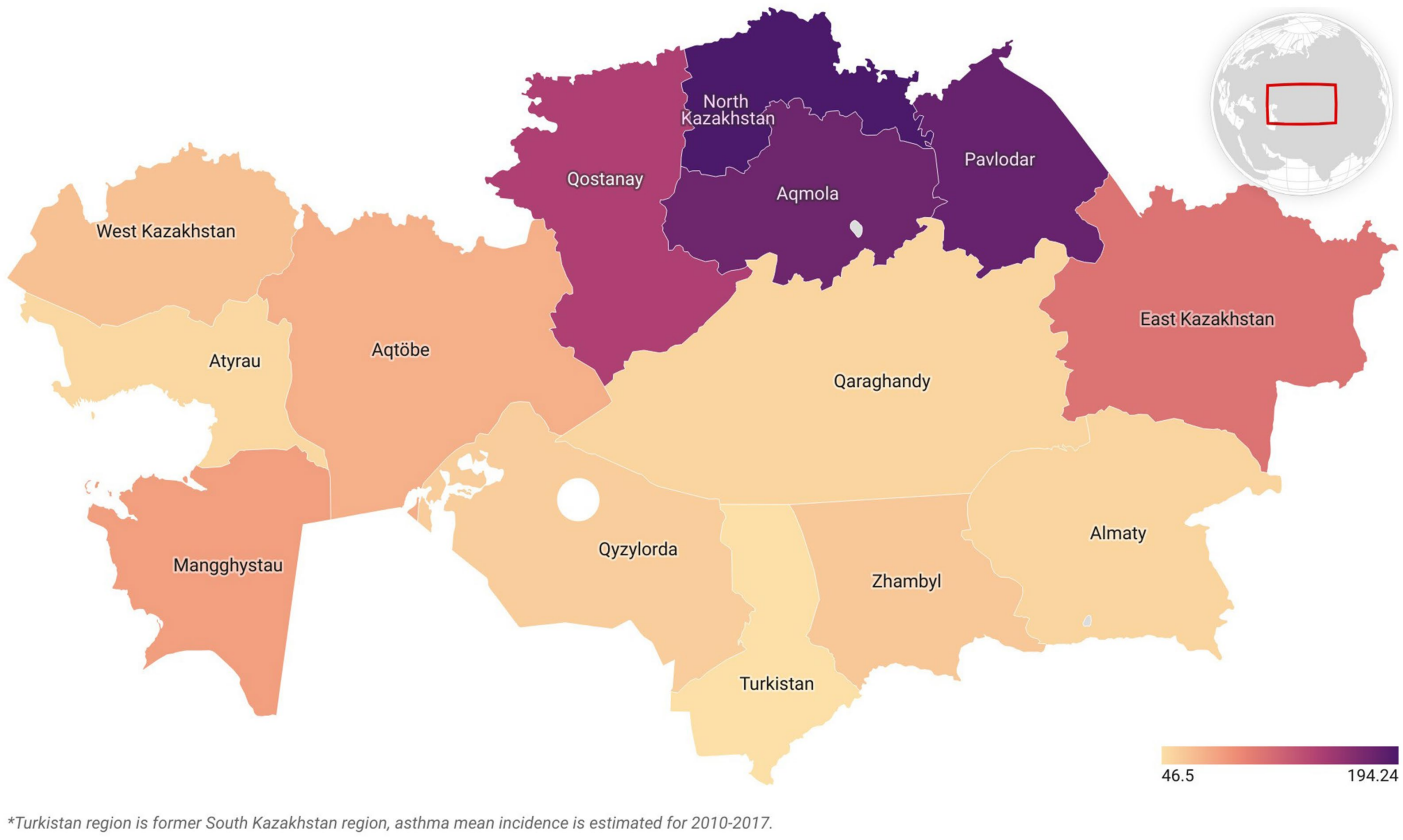
Mean asthma incidence across Kazakhstan regions, 2010–2019.

Asthma was selected for further analysis due to its pronounced increasing trend, with primary incidence rising from 46.5 to 194.2 per 100,000 children aged 0–14 years between 2010 and 2019—a 140.2% increase (Figure 1). The condition also showed high variability (CV = 0.72).

**Figure 2:**
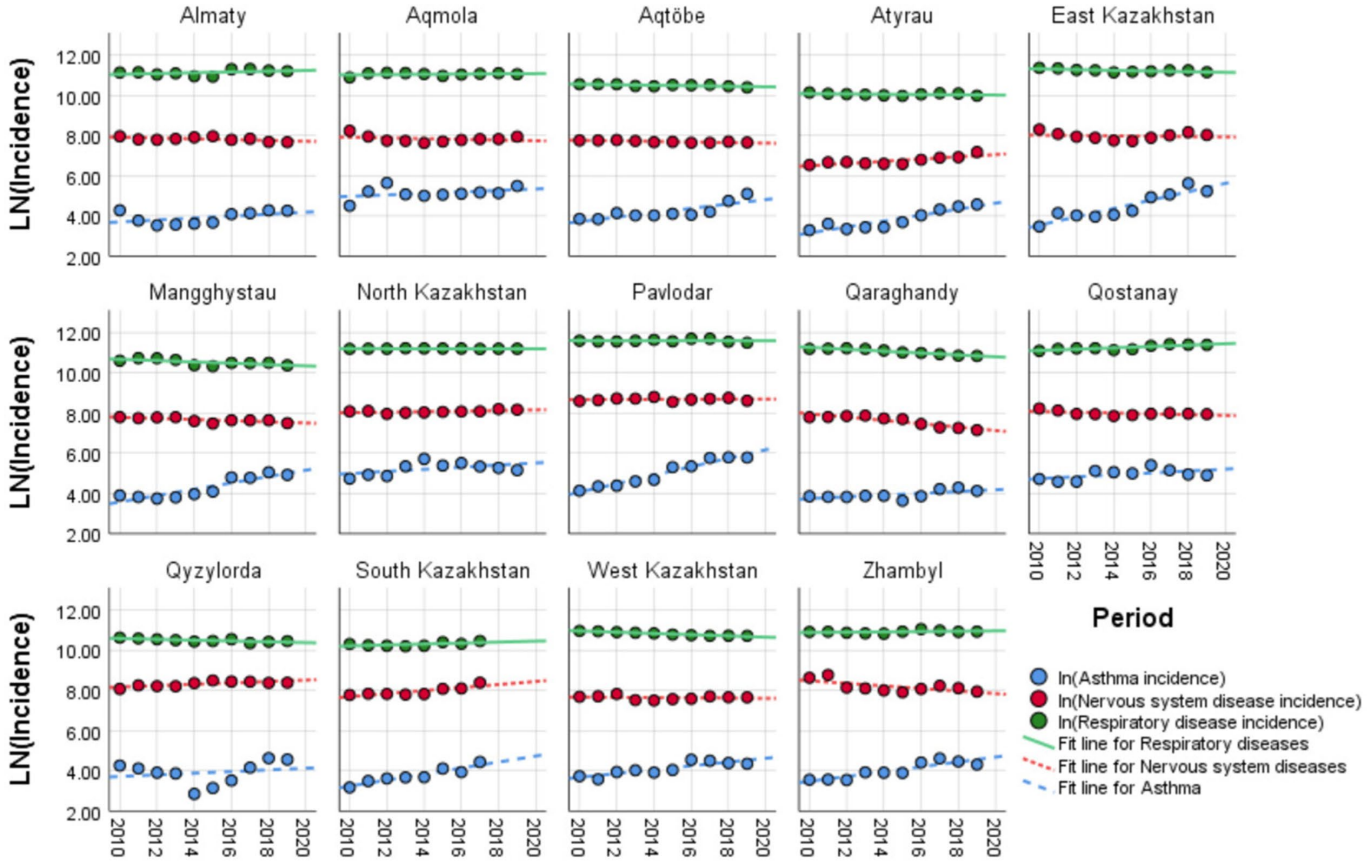
Temporal trends of disease incidence across Kazakhstan regions, 2010–2019: comparison of asthma, respiratory, and nervous system diseases.

In Figure 2, the log-transformed incidence trends of three pediatric conditions with their linear fit trends are displayed across 14 regions of Kazakhstan from 2010 to 2019. Regional variations were most evident for respiratory diseases, with significant differences across regions, while asthma and nervous system diseases showed limited regional heterogeneity. The incidence of nervous system disorders showed mixed patterns, with some regions remaining stable and others displaying slight increases or decreases over the decade.

### 3.2 Regression analyses

Spearman’s correlation analysis showed some associations between log-transformed disease incidence and socioeconomic/healthcare predictors. Respiratory diseases (J00–J99) had positive correlations with income inequality (Gini coefficient: *ρ* = 0.59, *p* < 0.01) and divorce rate (*ρ* = 0.73, *p* < 0.01), while showing negative associations with average salary (*ρ* = −0.33, *p* < 0.01) and GRP per capita (*ρ* = −0.27, *p* < 0.01). Asthma (J45) showed an inverse relationship with marriage rate (*ρ* = −0.68, *p* < 0.01) and pediatrician density (*ρ* = −0.59, *p* < 0.01), but correlated positively with housing provision (*ρ* = 0.55, *p* < 0.01) and living wage (*ρ* = 0.39, *p* < 0.01). Nervous system diseases (G00–G99) were inversely linked to GRP per capita (*ρ* = −0.49, *p* < 0.01) and positively associated with unemployment (ρ = 0.23, *p* < 0.01).

Before proceeding to the LMM, preliminary linear regression models were fitted for each log-transformed disease outcome (respiratory diseases, nervous system diseases, and asthma) using the set of log-transformed predictors to explore initial relationships. These log–log models provide elasticity estimates, where coefficients represent the percentage change in disease incidence associated with a 1% change in each predictor.

The linear regression model for respiratory diseases (adjusted *R*^2^ = 0.537) identified several significant predictors (Table 3). For instance, a 1% increase in the Gini coefficient was associated with a 1.50% increase in incidence (*p* < 0.001). Similarly, a 1% increase in housing provision per resident was associated with a 0.91% increase in incidence (*p* = 0.038). On the other hand, a 1% increase in the proportion of the population with income below the living wage was associated with a 0.20% decrease in incidence (*p* = 0.011). A 1% increase in population density corresponded to a 0.35% increase in incidence (*p* = 0.016).

**Table 3:** Linear regression results for respiratory diseases.

Predictor	Coefficient	95%CI	SE	*p*-value	VIF
LN(Gini)	1.5	1.09; 1.90	0.2	<0.001	1.40
LN(Income below living wage)	−0.2	−0.35; −0.05	0.08	0.011	1.79
LN(Housing per resident)	0.91	0.05; 1.76	0.43	0.038	1.74
LN(Population density)	0.35	0.07; 0.63	0.14	0.016	4.20
LN(GRP per capita)	−0.12	−0.27; 0.04	0.08	0.14	3.91
LN(Unemployed population)	−0.15	−0.39; 0.09	0.12	0.22	4.35
LN(Pediatrician density)	−0.06	−0.36; 0.25	0.15	0.72	2.81
LN(Hospital beds for children)	0.48	−0.03; 1.00	0.26	0.06	2.87
LN(Length of stay)	0.58	−0.25; 1.40	0.42	0.17	3.17

For nervous system diseases (adjusted *R*^2^ = 0.410), the linear regression model identified significant predictors (Table 4). A 1% increase in GRP per capita was associated with a 0.65% decrease in incidence (*p* < 0.001). In contrast, a 1% increase in the Gini coefficient was associated with a 0.12% increase in incidence (*p* < 0.001). There was a 1% increase in housing per resident, resulting in a 1.75% increase in incidence (*p* = 0.001).

**Table 4:** Linear regression results for nervous system diseases.

Predictor	Coefficient	95%CI	SE	*p*-value	VIF
LN(GRP per capita)	−0.65	−0.84; −0.46	0.10	<0.001	3.91
LN(Gini)	0.12	−0.36; 0.61	0.25	<0.001	1.40
LN(Housing per resident)	1.75	0.72; 2.78	0.52	0.001	1.74
LN(Population density)	−0.11	−0.45; 0.23	0.17	0.514	4.20
LN(Unemployed population)	−0.35	−0.65; −0.06	0.15	0.019	4.35
LN(Income below living wage)	−0.08	−0.27; 0.10	0.09	0.378	1.79
LN(Pediatrician density)	0.25	−0.12; 0.61	0.19	0.182	2.81
LN(Hospital beds for children)	0.71	0.09; 1.33	0.31	0.024	2.87
LN(Length of stay)	−1.22	−2.21; −0.23	0.50	0.016	3.17

The linear regression model for asthma (adjusted *R*^2^ = 0.525) identified several significant predictors (Table 5). In contrast, a 1% increase in the Gini coefficient was associated with a 1.68% increase in incidence (*p* < 0.001). A 1% increase in housing provision per resident was associated with a 4.23% increase in incidence (*p* < 0.001). There was a 1% increase in pediatrician density, resulting in a 0.76% decrease in incidence (*p* = 0.002).

**Table 5:** Linear regression results for asthma.

Predictor	Coefficient	95%CI	SE	*p*-value	VIF
LN(Gini)	1.675	−0.84; −0.46	0.32	<0.001	1.40
LN(Housing per resident)	4.227	2.89; 5.56	0.67	<0.001	1.74
LN(Pediatrician density)	−0.756	−1.23; −0.28	0.24	0.002	2.81
LN(GRP per capita)	−0.241	−0.49; 0.00	0.12	0.053	3.91
LN(Population density)	0.024	−0.42; 0.46	0.22	0.913	4.20
LN(Unemployed population)	−0.638	−1.02; −0.26	0.19	0.001	4.35
LN(Income below living wage)	0.337	0.10; 0.58	0.12	0.006	1.79
LN(Hospital beds for children)	−0.121	−0.92; 0.68	0.40	0.764	2.87
LN(Length of stay)	0.353	−0.93; 1.64	0.65	0.587	3.17

A LMM with a first-order autoregressive (AR1) covariance structure was fitted for the log-transformed incidence rate of respiratory, nervous system diseases, and asthma among children in Kazakhstan (2010–2019). Predictors included log-transformed GRP per capita, population density, unemployed population, Gini coefficient, income below living wage, housing per resident, pediatrician density, hospital beds for children, and length of stay. The model included fixed effects for “Region” (14 levels), “Period” (10 levels), and all predictors, a random intercept for “Region,” and an AR1 structure for repeated measures over “Period.”

The LMM for respiratory diseases identified significant fixed effects (Table 6). A 1% increase in population density was associated with a 1.048% decrease in incidence (*p* = 0.008). A 1% increase in the unemployed population was associated with a 0.406% increase in incidence (*p* = 0.029). A 1% increase in the Gini coefficient was associated with a 0.433% decrease in incidence (*p* = 0.003). A 1% increase in length of stay was associated with a 0.545% increase in incidence (*p* = 0.031). The “Region” variable was significant [*F*(13, 106) = 16.134, *p* < 0.001], with Zhambyl as the reference category. Aqtöbe (*p* = 0.002), Atyrau (*p* < 0.001), Mangghystau (*p* = 0.002), North Kazakhstan (*p* = 0.047), Pavlodar (*p* < 0.001), Qaraghandy (*p* = 0.033), Qyzylorda (*p* = 0.001), and South Kazakhstan (*p* < 0.001) differed significantly from Zhambyl. The “Period” was significant [*F*(9, 106) = 3.791, *p* < 0.001], with 2019 as the reference category. The year 2015 differed significantly from 2019 (*p* = 0.048). Non-significant predictors included GRP per capita (*p* = 0.902), income below living wage (*p* = 0.628), housing per resident (*p* = 0.262), pediatrician density (*p* = 0.223), and hospital beds for children (*p* = 0.666).

**Table 6:** Estimates of fixed effects for respiratory diseases.

Parameter	Estimate	SE	df	*t*	Sig.	95% CI
Intercept	10.652	1.511	106	7.051	0.000	7.657; 13.647
[Region = Almaty]	0.257	0.170	106	1.516	0.133	−0.079; 0.593
[Region = Aqmola]	−0.240	0.195	106	−1.231	0.221	−0.627; 0.147
[Region = Aqtöbe]	−1.250	0.390	106	−3.208	0.002	−2.023; −0.478
[Region = Atyrau]	−1.060	0.249	106	−4.252	0.000	−1.554; −0.566
[Region = East Kazakhstan]	−0.112	0.228	106	−0.492	0.624	−0.565; 0.340
[Region = Mangghystau]	−0.997	0.310	106	−3.215	0.002	−1.612; −0.382
[Region = North Kazakhstan]	0.345	0.172	106	2.010	0.047	0.005; 0.685
[Region = Pavlodar]	0.609	0.168	106	3.631	0.000	0.276; 0.941
[Region = Qaraghandy]	−0.790	0.366	106	−2.155	0.033	−1.516; −0.063
[Region = Qostanay]	−0.184	0.236	106	−0.781	0.437	−0.652; 0.284
[Region = Qyzylorda]	−1.085	0.319	106	−3.404	0.001	−1.718; −0.453
[Region = South Kazakhstan]	−0.682	0.189	106	−3.614	0.000	−1.056; −0.308
[Region = West Kazakhstan]	−0.450	0.247	106	−1.825	0.071	−0.939; 0.039
[Period = 2010]	−0.199	0.143	106	−1.393	0.167	−0.482; 0.084
[Period = 2011]	−0.135	0.127	106	−1.063	0.290	−0.386; 0.116
[Period = 2012]	−0.133	0.111	106	−1.198	0.233	−0.352; 0.087
[Period = 2013]	−0.125	0.092	106	−1.349	0.180	−0.308; 0.059
[Period = 2014]	−0.145	0.079	106	−1.836	0.069	−0.301; 0.012
[Period = 2015]	−0.144	0.072	106	−1.997	0.048	−0.287; −0.001
[Period = 2016]	−0.029	0.053	106	−0.547	0.585	−0.135; 0.077
[Period = 2017]	0.018	0.037	106	0.476	0.635	−0.056; 0.092
[Period = 2018]	0.019	0.023	106	0.827	0.410	−0.026; 0.064
ln_GRPCapita	0.010	0.081	106	0.123	0.902	−0.151; 0.171
ln_PopulationDensity	−1.048	0.385	106	−2.720	0.008	−1.812; −0.284
ln_Unemployed	0.406	0.184	106	2.212	0.029	0.042; 0.770
ln_Gini	−0.433	0.144	106	−3.010	0.003	−0.718; −0.148
ln_IncomeBelowLivingWage	−0.016	0.032	106	−0.486	0.628	−0.080; 0.048
ln_HousingPerResident	−0.279	0.247	106	−1.129	0.262	−0.769; 0.211
ln_PediatricianDensity	−0.089	0.073	106	−1.226	0.223	−0.234; 0.055
ln_HospitalBedsKids	0.064	0.147	106	0.433	0.666	−0.228; 0.355
ln_LOS	0.545	0.249	106	2.186	0.031	0.051; 1.039

The LMM for asthma showed significant fixed effects (Table 7). A 1% increase in income below living wage was associated with a 0.264% increase in incidence (*p* = 0.032). A 1% increase in length of stay was associated with a 1.896% increase in incidence (*p* = 0.045). Period was significant [*F*(9, 106) = 1.968, *p* = 0.050], with 2019 as the reference category. The years 2010 (*p* = 0.029), 2011 (*p* = 0.020), 2012 (*p* = 0.025), 2013 (*p* = 0.036), 2014 (*p* = 0.013), and 2015 (*p* = 0.022) differed significantly from 2019. Region was not significant overall [*F*(13, 106) = 1.489, *p* = 0.133], but North Kazakhstan (*p* = 0.029) and Pavlodar (*p* = 0.041) differed significantly from Zhambyl, the reference category. Non-significant predictors included GRP per capita (*p* = 0.814), population density (*p* = 0.695), unemployed population (*p* = 0.291), Gini coefficient (*p* = 0.582), housing per resident (*p* = 0.238), pediatrician density (*p* = 0.166), and hospital beds for children (*p* = 0.192).

**Table 7:** Estimates of fixed effects for asthma incidence.

Parameter	Estimate	SE	df	*t*	Sig.	95% CI
Intercept	4.115	5.648	106	0.729	0.468	−7.084; 15.314
[Region = Almaty]	−0.337	0.623	106	−0.541	0.589	−1.573; 0.898
[Region = Aqmola]	0.858	0.719	106	1.193	0.236	−0.568; 2.284
[Region = Aqtöbe]	0.004	1.450	106	0.003	0.998	−2.871; 2.880
[Region = Atyrau]	0.091	0.924	106	0.098	0.922	−1.741; 1.923
[Region = East Kazakhstan]	−0.196	0.846	106	−0.231	0.818	−1.872; 1.481
[Region = Mangghystau]	0.355	1.151	106	0.308	0.759	−1.928; 2.638
[Region = North Kazakhstan]	1.397	0.631	106	2.215	0.029	0.147; 2.647
[Region = Pavlodar]	1.272	0.616	106	2.066	0.041	0.051; 2.493
[Region = Qaraghandy]	−0.595	1.366	106	−0.436	0.664	−3.303; 2.112
[Region = Qostanay]	0.390	0.874	106	0.446	0.657	−1.343; 2.122
[Region = Qyzylorda]	−0.088	1.184	106	−0.075	0.941	−2.435; 2.258
[Region = South Kazakhstan]	−0.487	0.695	106	−0.700	0.486	−1.865; 0.892
[Region = West Kazakhstan]	0.021	0.912	106	0.023	0.982	−1.787; 1.829
[Period = 2010]	−1.185	0.535	106	−2.215	0.029	−2.246; −0.124
[Period = 2011]	−1.118	0.474	106	−2.358	0.020	−2.058; −0.178
[Period = 2012]	−0.941	0.415	106	−2.267	0.025	−1.763; −0.118
[Period = 2013]	−0.735	0.346	106	−2.123	0.036	−1.421; −0.049
[Period = 2014]	−0.748	0.296	106	−2.531	0.013	−1.335; −0.162
[Period = 2015]	−0.627	0.270	106	−2.321	0.022	−1.162; −0.091
[Period = 2016]	−0.261	0.200	106	−1.302	0.196	−0.657; 0.136
[Period = 2017]	−0.043	0.140	106	−0.308	0.758	−0.320; 0.234
[Period = 2018]	−0.021	0.085	106	−0.248	0.805	−0.190; 0.148
ln_GRPCapita	0.072	0.305	106	0.236	0.814	−0.533; 0.678
ln_PopulationDensity	−0.567	1.440	106	−0.394	0.695	−3.422; 2.288
ln_Unemployed	0.731	0.689	106	1.061	0.291	−0.635; 2.097
ln_Gini	0.298	0.540	106	0.552	0.582	−0.772; 1.369
ln_IncomeBelowLivingWage	0.264	0.122	106	2.168	0.032	0.023; 0.505
ln_HousingPerResident	−1.099	0.926	106	−1.186	0.238	−2.935; 0.738
ln_PediatricianDensity	−0.382	0.274	106	−1.396	0.166	−0.926; 0.161
ln_HospitalBedsKids	−0.723	0.552	106	−1.312	0.192	−1.817; 0.370
ln_LOS	1.896	0.935	106	2.028	0.045	0.042; 3.750

Zhambyl is the reference category for Region, and 2019 is the reference category for Period.

The final step for log-transformed nervous system disease incidence (Table 8) showed that a 1% increase in the unemployed population was associated with a 0.692% increase in incidence (*p* = 0.040). Region variable was not significant overall [*F*(13, 106) = 0.165, *p* = 1.000], with Zhambyl as the reference category, and no individual regions differed significantly from Zhambyl (all *p* ≥ 0.953). Period was not significant [*F*(9, 101.622) = 1.106, *p* = 0.366], with 2019 as the reference category, and no individual years differed significantly from 2019 (the *p*-values for all the individual years ≥0.388). Most of the predictors were non-significant, including GRP per capita (*p* = 0.921), population density (*p* = 0.971), Gini coefficient (*p* = 0.963), income below living wage (*p* = 0.990), housing per resident (*p* = 0.979), pediatrician density (*p* = 0.938), hospital beds for children (*p* = 0.816), and length of stay (*p* = 0.548).

**Table 8:** Estimates of fixed effects for nervous system disease incidence.

Parameter	Estimate	SE	df	*t*	Sig.	95% CI
Intercept	5.750	3.163	1.037	1.818	0.313	−31.218; 42.718
[Region = Almaty]	−0.845	1.585	0.018	−0.533	0.964	−3.998, 2.308
[Region = Aqmola]	−0.056	1.599	0.018	−0.035	0.995	−2.243; 2.243
[Region = Aqtöbe]	−0.367	1.741	0.026	−0.211	0.973	−2.669; 2.669
[Region = Atyrau]	−0.940	1.629	0.020	−0.577	0.959	−2.527; 2.526
[Region = East Kazakhstan]	−0.323	1.614	0.019	−0.200	0.978	−2.410; 1.626
[Region = Mangghystau]	−0.153	1.677	0.022	−0.091	0.987	−2.740; 1.147
[Region = North Kazakhstan]	0.198	1.588	0.018	0.125	0.985	−2.635; 1.263
[Region = Pavlodar]	0.550	1.586	0.018	0.347	0.971	−2.706; 2.706
[Region = Qaraghandy]	−0.969	1.711	0.024	−0.566	0.953	−3.701; 2.902
[Region = Qostanay]	−0.187	1.621	0.019	−0.115	0.986	−2.827; 1.808
[Region = Qyzylorda]	0.271	1.686	0.023	0.161	0.979	−2.482; 1.724
[Region = South Kazakhstan]	−0.691	1.596	0.018	−0.433	0.966	−2.351; 2.782
[Region = West Kazakhstan]	−0.241	1.632	0.020	−0.148	0.982	−2.445; 2.173
[Period = 2010]	−0.061	0.270	98.974	−0.225	0.822	−0.597; 0.476
[Period = 2011]	−0.045	0.240	98.973	−0.187	0.852	−0.521; 0.432
[Period = 2012]	−0.109	0.210	99.853	−0.518	0.605	−0.525; 0.307
[Period = 2013]	−0.122	0.175	100.557	−0.697	0.488	−0.468; 0.225
[Period = 2014]	−0.123	0.149	101.873	−0.827	0.410	−0.418; 0.172
[Period = 2015]	−0.117	0.135	104.160	−0.867	0.388	−0.384; 0.150
[Period = 2016]	−0.052	0.100	104.603	−0.521	0.603	−0.249; 0.146
[Period = 2017]	0.020	0.069	105.306	0.290	0.772	−0.117; 0.157
[Period = 2018]	0.019	0.041	105.869	0.451	0.653	−0.063; 0.100
ln_GRPCapita	−0.015	0.146	103.933	−0.100	0.921	−0.304; 0.274
ln_PopulationDensity	−0.028	0.766	79.510	−0.036	0.971	−1.552; 1.497
ln_Unemployed	0.692	0.333	105.766	2.078	0.040	0.032; 1.353
ln_Gini	0.012	0.255	102.150	0.046	0.963	−0.495; 0.518
ln_IncomeBelowLivingWage	0.001	0.057	101.907	0.012	0.990	−0.113; 0.114
ln_HousingPerResident	−0.012	0.463	103.732	−0.027	0.979	−0.931; 0.906
ln_PediatricianDensity	−0.010	0.131	103.479	−0.078	0.938	−0.269; 0.249
ln_HospitalBedsKids	−0.061	0.263	103.772	−0.233	0.816	−0.583; 0.461
ln_LOS	0.273	0.453	105.397	0.602	0.548	−0.625; 1.171

Zhambyl is the reference category for Region, and 2019 is the reference category for Period.

In summary, the respiratory diseases were the most prevalent, affecting over 57,000 per 100,000 children annually in a study period. Respiratory diseases were strongly linked to socioeconomic factors. Higher unemployment and longer hospital stays were associated with increased incidence, while a greater income was linked to a slight decrease, possibly due to better healthcare access in wealthier regions. Asthma incidence was higher in areas with more poverty and longer hospital stays, with significant increases observed over time, particularly in regions like North Kazakhstan and Pavlodar. Nervous system diseases, though less prevalent, were tied to higher unemployment. We identified regional differences, with respiratory diseases varying significantly across areas like Atyrau and Pavlodar, while asthma showed a consistent upward trend nationwide.

## 4 Discussion

This study specifically examines the pre-COVID-19 era (2010–2019), a period before the global pandemic significantly altered health indicators through both direct and indirect effects. The COVID-19 pandemic, starting in 2020, introduced unprecedented disruptions, including overwhelmed healthcare systems, delayed routine care, and socioeconomic shifts due to lockdowns, which impacted disease incidence and healthcare indicators worldwide (34, 35). For instance, a study from Italy found that pediatric emergency visits for respiratory illnesses dropped sharply during the pandemic due to reduced social contact, while Barbiellini Amidei et al. (36) and Santoli et al. (37) reported a decline in routine vaccinations in the U.S., potentially increasing vulnerability to other diseases. In Kazakhstan, where healthcare resources were already strained with public health spending at 1.8–3.2% of GRP, the pandemic had complex effects on healthcare financing (24). While Shaltynov et al. (38) documented a decrease in out-of-pocket expenditures during 2020–2021, with catastrophic health expenditure incidence declining nearly twofold to 1.32 and 1.24% respectively, this occurred alongside reduced access to non-COVID care, suggesting potential unmet healthcare needs rather than improved financial protection. This interpretation is further supported by evidence that active tuberculosis detection in Kazakhstan decreased by 17–20% during the pandemic (35), indicating significant disruptions to essential public health services (39). By focusing on the pre-COVID-19 period, our study provides a baseline understanding of pediatric disease incidence, capturing patterns of respiratory diseases, asthma, and nervous system diseases among children aged 0–14 years without the confounding effects of the pandemic.

The high burden of respiratory diseases among children in Kazakhstan mirrors global patterns observed in other countries, with relatively stable incidence trends punctuated by periodic increases (40, 41). Beyond direct environmental exposures, our findings align with international literature reporting significant associations between pediatric respiratory outcomes and broader socioeconomic and healthcare indicators (42). We found that a 1% increase in population density was associated with a 1.048% decrease in respiratory disease incidence (*p* = 0.008). This finding contrasts with studies in Western countries where higher population density often correlates with increased respiratory morbidity due to urban pollution and overcrowding (43). However, it is important to recognize the mean population density is very low in Kazakhstan, with 5.8 people per 1 sq. km. (44). Moreover, this inverse relationship may reflect the centralized healthcare infrastructure in urban centers such as Astana, Almaty, and Shymkent, which offer better access to pediatric hospitals and early diagnosis, thereby mitigating environmental risks. It could be assessed in future studies as an interesting case to explore.

While Alam et al. (45) demonstrated that in the Eastern Mediterranean Region, a unit increase in the Gini coefficient was associated with approximately 7.2 and 3.9% increases in COVID-19 cases and deaths per million population, respectively, our study shows an inverse relationship: a 1% increase in the Gini coefficient was linked to a 0.433% decrease in respiratory disease incidence (*p* = 0.003). This finding diverges from typical associations where income inequality aggravates health disparities (46). The unexpected inverse relationship between the Gini coefficient and respiratory disease incidence may reflect regional dynamics where higher income inequality coincides with concentrated wealth in resource-rich regions like Atyrau, potentially enabling investments in healthcare infrastructure that improve disease detection and management. Also, this finding could indicate underreporting in less equitable regions with weaker health systems, masking true disparities. It’s critical to acknowledge that the Gini coefficient, while widely used, simplifies the income distribution into a single metric, potentially obscuring nuanced differences in living standards between different areas (47, 48). A 1% rise in unemployment correlated with a 0.406% increase in incidence (*p* = 0.029), while longer hospital stays (+0.545%, *p* = 0.031) likely reflect respiratory disease severity. Unemployment’s impact mirrors findings in post-Soviet economies, where job loss exacerbates household stressors and worsens well-being (49, 50). Infants and young children, even those without chronic or serious underlying medical conditions, are at elevated risk for hospitalization during influenza seasons (51). However, the burden is disproportionately higher among older children with asthma, younger children with lower respiratory infections, those with chronic comorbidities, and children hospitalized in large urban hospitals (52). Moreover, insufficient infrastructure in Kazakhstan impedes the detection of less prevalent diseases such as interstitial lung disease, along with vigilance from primary healthcare providers (53).

We identified the 140% rise in pediatric asthma incidence in Kazakhstan within the study period. It may reflect a broader “double burden” in transitional economies, where industrialization and urbanization amplify NCD risks even as infectious diseases persist. Similar trends were reported in Azerbaijan, Ukraine, where post-Soviet transitions in the economy correlated with an asthma patients increase in children and adult populations (54). Our finding that income inequality drives asthma (+0.26% per 1% rise in low-income households) aligns with evidence from systematic reviews suggesting that asthma is associated with lower socioeconomic position. This highlights how economic disadvantage contributes to respiratory vulnerability through multiple pathways, including inadequate housing conditions, reduced access to healthcare, and greater exposure to environmental triggers (55, 56).

The role of unemployment in our models (+0.41% for respiratory diseases, +0.69% for nervous system disorders) suggests economic instability erodes protective factors like nutrition and parental caregiving, a mechanism observed in neighboring Russia during the 2000s, where maternal unemployment was associated with 3.4-fold higher odds of childhood asthma (57).

The potential for inter-disease effects, such as the influence of other conditions like digestive or blood disorders on the selected diseases, was not directly modeled due to data aggregation. However, asthma was analyzed separately from the broader respiratory disease category (J00–J99) to avoid collinearity, as it is a subset of respiratory conditions (J45). Future studies could incorporate other diseases as covariates to explore indirect effects.

The limited associations observed for nervous system disorders in our study, with unemployment being the sole significant predictor, contrast with findings from high-and middle-income countries, where multiple socioeconomic factors typically correlate with neurological outcomes. Robust evidence demonstrates that lower socioeconomic status, including unemployment, is associated with a higher burden of pediatric neurological conditions (58, 59). These disparities likely stem from differentials in healthcare access, educational resources, and early diagnostic capacity (60). Our findings suggest Kazakhstan’s neurological health patterns may reflect either systemic under-detection of cases across socioeconomic strata, potentially due to limited specialist availability in some regions, or a more homogeneous distribution of neurological risk factors across population groups compared to other national contexts.

In Kazakhstan, ongoing reforms have focused on transitioning from a centralized, hospital-based healthcare model inherited from the Soviet era to a socially oriented, people-centered PHC system with the aim to achieve universal health coverage (61). Extensive sanitary and anti-epidemic measures at the national level, along with supervision of childcare institutions, led to a drop in childhood morbidity and mortality in all country regions (62). Key challenges remain, such as the overall lack of public funding for primary care, poor financial protection, access to primary care in rural areas, and underdeveloped quality monitoring (63).

Based on our findings, to tackle the elevated incidence of respiratory and nervous system diseases in high-unemployment regions such as Pavlodar region, where economic constraints limit healthcare access, we see that there could be better deployment of mobile health units. These units would be equipped with pediatric specialists and diagnostic tools to deliver timely care to underserved communities, thereby reducing disease burden and improving early intervention. Strengthening socioeconomic support programs, including nutritional subsidies and community health worker initiatives, can alleviate financial barriers and promote healthier living conditions, particularly in rural areas (64, 65). In regions characterized by high income inequality, such as Atyrau, and mostly rural regions like Zhambyl, where disparities may play a role in asthma incidence due to environmental and economic factors, we advocate for the implementation of targeted asthma prevention programs. Access to and affordability of essential inhaled asthma drugs are major challenges to effective asthma control in many countries (66). These programs could include environmental monitoring, air quality regulation, and subsidies for asthma medications and devices, alongside educational campaigns targeting vulnerable populations (67, 68).

This study has several limitations that should be taken into account. First, the use of regionally aggregated data may obscure intra-regional variations or individual-level factors, limiting granularity. Due to the use of regionally aggregated data, individual-level variables were unavailable, limiting our ability to analyze age-specific variations. While the data underwent standard validation, regional underreporting bias may exist, potentially affecting the accuracy of incidence rates, and other selected variables. Second, the unavailability of data from South Kazakhstan after 2017, due to its administrative reorganization, may have impacted the analysis of regional variations when using pooled data from 2010 to 2019. This limitation could reduce the precision of estimates for this region and affect the overall assessment of regional heterogeneity. Third, the LMMs, while accounting for clustering and autocorrelation, may not capture all sources of variability, such as unmeasured environmental factors (ambient air pollution, indoor air quality, climate indicators, etc.). These environmental determinants are particularly important for respiratory conditions such as asthma, as substantial evidence links air pollutants and climate variables to respiratory symptom exacerbation and disease progression in pediatric populations (11, 69). For example, our findings show that the significantly lower incidence of respiratory diseases in industrial regions such as Aqtöbe and Atyrau contrasts with the higher burden in coal-dependent Pavlodar. The absence of these environmental metrics represents a significant gap, especially for Kazakhstan’s industrial regions, where such exposures may disproportionately affect respiratory health outcomes. However, consistent reliable regional data for air pollutants were not available prior to 2019, though recent advancements in environmental monitoring systems will enable future studies to incorporate these key parameters into statistical models. Also, we did not have some parameters, such as cases with comorbidities, parental education status, cultural aspects that may influence greatly children health outcomes (70–72). Finally, the log-transformation assumes proportional relationships, which may not hold for all predictors, potentially affecting elasticity estimates.

## 5 Conclusion

Using panel data from 14 regions and log-transformed linear mixed models, this study identified distinct socioeconomic, demographic, and healthcare predictors of respiratory diseases, asthma, and nervous system diseases incidence. Respiratory diseases and asthma in Kazakhstani children aged 0–14 appear closely linked to regional economic conditions, healthcare access, and inequality. Population density and income inequality were found as consistent predictors, while nervous system disorders showed weaker associations. Regional patterns in child morbidity reflect underlying socioeconomic disparities. Addressing socioeconomic disparities through place-based policies may prove effective in reducing the burden of pediatric respiratory and nervous system diseases, particularly in regions with greater income inequality and limited healthcare access. Addressing inequality and improving healthcare access may reduce the burden of pediatric disease and advance equity in health policy within the post-Soviet context.

## Data Availability

Publicly available datasets were analyzed in this study. This data can be found: data were derived from public domain resources: agency for Strategic planning and reforms of the Republic of Kazakhstan Bureau of National statistics—Main. Available online at: https://stat.gov.kz/en/.
